# Hydrogen peroxide disrupts the regulatory pathway of saliva secretion in two salivary acinar rat cell lines

**DOI:** 10.3389/fmolb.2024.1480721

**Published:** 2024-11-13

**Authors:** Golnaz Golnarnik, Tine M. Søland, Hilde K. Galtung, Trude M. Haug

**Affiliations:** Department of Dentistry, Institute of Oral Biology, University of Oslo, Oslo, Norway

**Keywords:** salivary gland, oxidative stress, Ca^2+^ signaling, reactive oxygen species, submandibular gland, parotid gland

## Abstract

**Background:**

Secretion of saliva is controlled by autonomic nerve signals via regulation of Ca^2+^-dependent ion transport across acinar cell membranes. Oxidative stress may affect this process, leading to a decrease in saliva production. This study investigates elements of the Ca^2+^ regulatory pathway and their vulnerability to hydrogen peroxide-induced oxidative stress.

**Methods:**

Rat parotid and submandibular salivary gland acinar cell lines were exposed to different hydrogen peroxide concentrations to simulate oxidative stress. Cell viability and intracellular reactive oxygen species were measured, mRNA levels were assessed via RT-qPCR, and protein expression was studied using western blot and immunofluorescence microscopy.

**Results:**

Elevated concentrations of hydrogen peroxide reduced cell viability and increased intracellular levels of reactive oxygen species and led to a decrease in cholinergic receptor muscarinic 3 and adrenoreceptor alpha 1A mRNA and protein levels in both cell lines. In parotid gland cells, both mRNA and protein levels of stromal interaction molecule 1 and Orai1 decreased with increasing concentrations of hydrogen peroxide. In contrast, in submandibular gland cells stromal interaction molecule 1 and Orai1 displayed differential mRNA and protein expression levels.

**Conclusion:**

Our study revealed that hydrogen peroxide exposure alters rat parotid and submandibular acinar cells, increasing reactive oxygen species and reducing autonomic receptor expression. Differential mRNA and protein expression of stromal interaction molecule 1 and Orai1 highlight complex oxidative stress effects on Ca^2^⁺ signaling. Most likely these effects will be deleterious to salivary secretion, but some effects may be protective.

## Introduction

Saliva is produced by the major salivary glands, including the parotid glands (PG), submandibular glands (SMG), and sublingual glands (SL), in addition to hundreds of minor salivary glands (Proctor, 2016). It plays a crucial role in maintaining oral hygiene, as well as in facilitating chewing and swallowing, protecting the oral cavity, and solubilizing food for taste. Hyposalivation may lead to oral discomfort, speech and swallowing difficulties, altered taste, and increased risk of oral infections, all significantly affecting quality of life. Furthermore, hyposalivation represents a challenge for patients receiving head and neck cancer radiation, as well as in patients with Sjögren’s syndrome, diabetes, and the aging population as a whole. Hyposalivation under all these conditions may be partially linked to increased oxidative stress (Ambudkar, 2018; Matsumoto et al., 2021; Santos et al., 2023).

Acinar cells, located in the salivary gland acini, synthesize and secrete primary saliva (Proctor, 2016). Fluid secretion regulation involves elevation of cytosolic Ca^2+^ levels and requires coordinated activity of receptors, ion channels, and transporters. Parasympathetic nerves release acetylcholine and stimulate fluid and electrolyte secretion via M-muscarinic receptors while sympathetic nerves release noradrenaline, activating alpha adrenoreceptors for fluid secretion. The M3-muscarinic receptor (M3R or Chrm3) and alpha-1A adrenoreceptor (α1-AR or Adra1a) are both G-protein-coupled, and trigger Ca^2+^ release from the endoplasmic reticulum (ER) via inositol 1,4,5-trisphosphate receptors (IP_3_R) (Proctor and Carpenter, 2007; Ambudkar, 2014). This rise in cytosolic Ca^2+^ activates ion channels and pumps, forming an osmotic gradient for water secretion through aquaporin 5 (Aqp5), which is also regulated by Ca^2+^ (D'Agostino et al., 2020). However, the main mechanism for prolonged elevation of cytosolic Ca^2+^ is Store-Operated Ca^2+^ Entry (SOCE). Under normal physiological conditions, SOCE is activated in response to the depletion of Ca^2+^ stores in ER induced by IP_3_. In salivary gland cells, SOCE is mediated by the plasma membrane Ca^2+^ channel Orai1 (sometimes also transient receptor potential cation channel 1 (TRPC1), activated by stromal interaction molecule 1 (STIM1). STIM1 is a Ca^2+^ binding protein located in the ER membrane that detects decreases in ER-Ca^2+^ levels, and activates Orai1 to allow Ca^2+^ entering the cytosol to subsequently refill the ER store via the Ca^2+^ ATPase SERCA (Ambudkar, 2014; Ambudkar, 2016).

**FIGURE 1:**
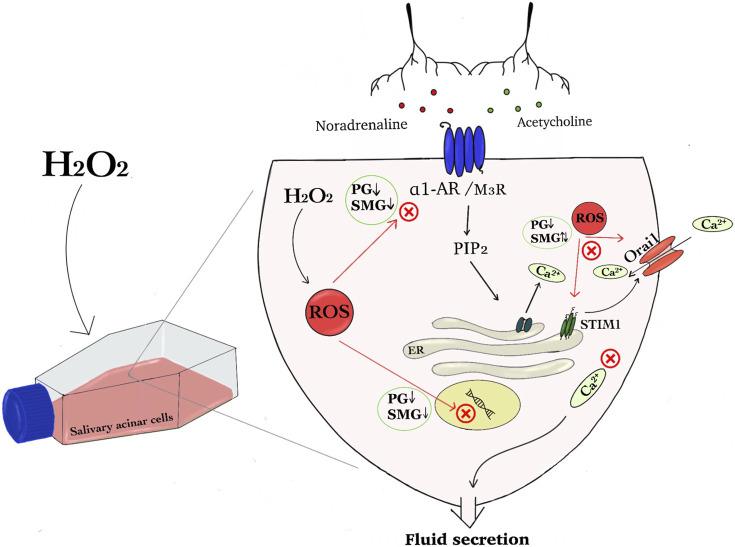
The graphical abstract summarises the effect of hydrogen peroxide on regulatory components of the Ca^2+^ signaling pathway in salivary acinar cells. We show that H₂O₂ induced increased amount of reactive oxygen species (ROS) in parotid gland (PG) and submandibular gland (SMG) acinar cells and hypothesize that this disrupts cellular signaling that induce fluid secretion. Protein and mRNA expression analysis indicated that H₂O₂ exposure will interfere with neurotransmitter signaling pathways involving noradrenaline and acetylcholine, since the expression of both receptors was reduced in both PG and SMG cells. Additionally, H₂O₂ impacted Store-Operated Calcium Entry (SOCE) components, specifically Orai1 and STIM1, leading to differential expression changes in the two glands. This dysregulation can affect Ca^2^⁺ homeostasis and ion transport, and subsequently fluid secretion (Abbreviations: hydrogen peroxide (H_2_O_2_), reactive oxygen species (ROS), parotid gland (PG), submandibular gland (SMG), α1 adrenergic receptor (α1-AR) and muscarinic receptor 3 (M3R), Store-Operated Calcium Entry (SOCE), stromal interaction molecule 1 (STIM1), Phosphatidylinositol 4,5-bisphosphate (PIP2) and Inositol 3-phosphate (IP3)).

Reactive oxygen species (Viswanathan, #16) range from highly reactive molecules including OH· (hydroxyl radical) to longer-lived, membrane-permeable ones such as hydrogen peroxide (H_2_O_2_). A moderate ROS increase, or oxidative eustress, is a normal, necessary cellular response, serving as an intracellular signal. In contrast, excessive ROS levels turn eustress into oxidative distress, causing cellular damage and dysfunction (Niki, 2016). Intracellular ROS increases due to stressors such as radiation, inflammation, and mitochondrial aging (Checa and Aran, 2020). Increased oxidative stress can modify SOCE, affecting cytosolic and ER Ca^2+^ levels (Nunes and Demaurex, 2014). For instance, it has been shown that radiation exposure of salivary glands activates a ROS-sensitive plasma membrane channel, enabling Ca^2+^ influx (Liu et al., 2017). This influx increases mitochondrial ROS, activates caspase 3, cleaves STIM1, and reduces SOCE in acinar cells. Nonetheless, the exact biological mechanism of oxidative stress-induced hyposalivation remains unclear. This study investigated how increased oxidative stress affects specific components of the Ca^2+^ signaling system in a parotid and a submandibular rat salivary gland acinar cell line (Figure 1).

## Materials and methods

### Cell culture

The immortalized acinar epithelial cell lines from parotid gland (PG C10) and submandibular gland (SMG C10) of sexually mature male Sprague Dawley rats were a kind gift from Dr. David Quissell at the University of Colorado, United States (Quissell et al., 1997; Quissell et al., 1998). Cells were cultured in Dulbecco’s Modified Eagle Medium/Nutrient Mixture F-12 (DMEM/F12 50:50) (Gibco, Thermo Fisher Scientific, Massachusetts, United States), supplemented with 5 mM L-glutamine, 4 mg/mL insulin, 2.5% fetal bovine serum (FBS), 0.8 mg/mL epidermal growth factor (EGF), 0.1 µM retinoic acid, 10 mg/mL hydrocortisone, 5 mg/ml T3 (3,3′,5-triiodo-L-thyronine sodium salt), 1‰ trace element mix (100x) (BioSource International, Camarillo, California, United States) and 50 μg/mL antibiotic (gentamicin) in a humidified atmosphere of 5% CO_2_ in air at 37°C. Unless otherwise noted, all chemicals were from Sigma-Aldrich (St. Louis, MO, United States). The experiments were performed on cells from passages 6-9.

### Exposure to H_2_O_2_

**FIGURE 2:**
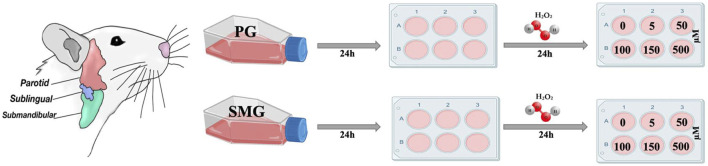
Graphical overview of the experimental design.

H_2_O_2_ was added from 5, 50, 500 Mm stock solutions in 5 mL of the normal growth medium to achieve final concentrations of 5, 50, 100, 150, and 500 µM. PG and SMG cells were seeded into 6-well plates at a density of 1.5 × 10^5^ cells/well and kept overnight before the growth medium was replaced with a new medium containing the different concentrations of H_2_O_2_. Cells used for negative control were given new medium without addition of H_2_O_2_. The cells were then incubated for 24 h before the experiment (Figure 2).

### Determination of cell viability

To assess cell viability after H_2_O_2_ treatment, a Trypan blue dye exclusion test was conducted after 24 h. Initially, 500 μL of 37°C trypsin-EDTA was added to each well, incubating for 10 min to detach cells. After deactivating trypsin with 1.5 mL of culture medium and centrifuging (Heraeus Megafuge 1.0R Refrigerated Centrifuge, Thermo Fisher Scientific, Massachusetts, United States) at 1000 RPM for 5 min, the supernatant was discarded, and the pellet resuspended in 1.5 mL medium. Then, 5 μL Trypan Blue Stain (0.4%) (Invitrogen, Thermo Fisher Scientific, Massachusetts, United States) was mixed with 500 μL cell suspension, and 10 μL of this mixture was placed in Chamber Slides (Invitrogen, Thermo Fisher Scientific, Massachusetts, United States) for analysis with the Countess II Automated Cell Counter (Invitrogen, Thermo Fisher Scientific, Massachusetts, United States), identifying viable/dead cells by size, circularity, and brightness. This procedure was repeated with five biological replicates.

### RT-qPCR

After 24 h treatment with different H_2_O_2_ concentrations, total RNA was isolated using RNeasy Mini Kit (Qiagen, Hilden, Germany), and reverse transcriptions were performed using the Reverse Transcriptase Core Kit (Eurogentec, Seraing, Belgium). qRT-PCR was conducted using AriaMx Real-Time PCR System (Agilent Technologies, Santa Clara, California, United States). Each reaction mixture consisted of 10 μL master mix (Low ROX MasterMix dTTP Blue, Takyon, Eurogentec), 10 μL cDNA, 3 μL nuclease-free water, 2 μL TaqMan® probes: *Orai1* (Assay ID: Rn02397170_m1), *Stim1* (Assay ID: Rn01506495_m1), *Adra1a* (Assay ID: Rn00567876_m1), and *Chrm3* (Assay ID: Rn00560986_s1). The large ribosomal subunit protein eL27 (*RPL27*) was used as a control (Applied Biosystems, Thermo Fisher Scientific, California, United States). Comparative quantification of expression was performed using the 2^−ΔΔCT^ method. RT-qPCR was conducted with five biological and three technical replicates for each probe.

### Intracellular ROS measurements

Intracellular oxidative stress was analyzed using the ROS-sensitive probe 5-(and-6-)-chloromethyl-2’,7’-dichlorodihydrofluorescein diacetate, acetyl ester (CM-H2DCFDA, Invitrogen, Thermo Fisher Scientific, Massachusetts, United States), with three technical replicates per sample. Cells (4 × 10^4^/well) were seeded in a 96-well black plate and incubated overnight. After removing the medium, 1 µM CM-H2DCFDA was added and incubated at 37°C for 30 min in darkness. Next, the CM-H2DCFDA solution was removed, and 100 µL of cell medium containing different H_2_O_2_ concentrations (0, 5, 50, 100, 150, and 500 µM) was added to each well. During 60 min, DCF (2′, 7′-dichlorofluorescein) fluorescence intensity was measured using a Cytation 3 fluorescence microplate reader (Agilent BioTek, California, United States).

### Western blot

As RT-qPCR showed the most significant changes at high H_2_O_2_ concentrations, Western blot was performed only with 100 and 150 µM H_2_O_2_. Parotid and submandibular gland acinar cells were exposed 24 h to 100 and 150 µM H_2_O_2_ concentrations, and untreated cells were used as controls. After removing the medium, the cells were washed with 1 mL PBS, and 500 μL of 37°C trypsin-EDTA was added to each well for 10 min to detach cells. After deactivating trypsin with 1.5 mL of culture medium and centrifuging (Heraeus Megafuge 1.0R Refrigerated Centrifuge, Thermo Fisher Scientific, Massachusetts, United States) at 1,000°RPM for 5 min, the supernatant was discarded. The pellet was resuspended in 100 μL CelLytic^™^ MT Cell Lysis Reagent (Sigma-Aldrich). Then, 1 μL EDTA and protease inhibitors (Thermo Fisher Scientific, Rockford, IL, United States) were added and mixed with a homogenizer every 10 min for at least 30 min while kept on ice. The lysed samples were centrifuged for 15 min at 13,000 rpm to remove insoluble material. The BioRad protein assay (Bio-Rad, Hercules, CA, United States) was used to determine total protein concentrations using gamma-globulin as a standard. Total protein (35 μg) was mixed with SDS sample buffer and PBS and heated to 70°C for 5 min. Equal sample volumes were loaded in each well in a Bolt^™^ Bis-Tris Plus Mini Protein Gels, 4%–12%, 1.0 mm, WedgeWell^™^ format (Invitrogen, Carlsbad, CA, United States). After gel electrophoresis, proteins were transferred to 0.45 μm Immobilon®-P PVDF Membrane (Merk, Darmstadt, Germany). The membranes were blocked for non-specific binding with casein blocking buffer (Sigma Aldrich, Darmstadt, Germany) over night. Next, membranes were incubated overnight with unlabeled rabbit anti-Orai1 (Alomone Labs, Israel), mouse anti-Stim1 (Alomone Labs), rabbit anti-Chrm3 (Bioss, Massachusetts, United States), rabbit anti-alpha1a adrenergic receptor (Invitrogen, Thermo Fisher Scientific, Massachusetts, United States), mouse anti-beta actin (Proteintech, United States) at 4°C. Beta-actin was used as a loading control. The membranes were washed in Tris buffered saline with Tween® 20 (TBST) (Sigma Aldrich, St. Louis, MO, United States) and incubated in secondary antibody for 1 h at room temperature. After additional washing in TBST, the membranes were incubated for 10 min in 1-Step^™^ NBT/BCIP Substrate Solution (Thermo Fisher Scientific, Rockford, IL, United States).

### Immunocytofluorescence staining

Untreated control cells and cells exposed to 100 and 150 μM H₂O₂ were seeded on 13 mm glass coverslips (VWR®, Pennsylvania, United States) at a density of 250,000 cells per well in a 24-well plate. The cells were incubated for 24 h, then fixed in 4% paraformaldehyde for 10 min at room temperature. After washing with PBS, cells were blocked in bovine serum albumin (BSA) 1% overnight. Before staining, cells were permeabilized with 0.1% Triton-X100 in 0.1% sodium citrate (w/v), then blocked with Avidin 10 μg/mL for 30 min. After washing with PBS, cells were blocked with Biotin 1 μg/mL for 30 min. Next, the coverslips were immersed in 5% normal horse serum (ThermoFisher Scientific, Massachusetts, United States) and incubated overnight with 2 μg/mL unlabeled rabbit anti-Orai1 (Alomone Labs, Israel) and 2 μg/mL unlabeled mouse anti-Stim1 (Santa Cruz Biotechnology, Dallas, TX, United States) at 4°C. After washing, cells were incubated with biotinylated horse anti-mouse IgG (Vector Laboratories), donkey anti-rabbit IgG (ThermoFisher Scientific), and Cy3-conjugated streptavidin (ThermoFisher Scientific). Nuclei were stained with 4’,6-diamidino-2-phenylindole (DAPI, ThermoFisher Scientific). The coverslips were mounted with a polyvinyl alcohol mounting medium containing DABCO (1,4-diazabicyclo [2.2.2] octane). Photographs were taken using a Nikon E90i microscope equipped with DS-Ri1 camera using NIS-elements software (Nikon Instruments Europe, Amstelveen, Netherlands). The photographs were analyzed using Adobe Photoshop CS6. The fluorescence intensity was measured in nine areas of images with ImageJ.

### Statistical analysis

Statistical analyses were performed using GraphPad Prism for Windows (https://www.graphpad.com/; version 9.5.1) and Excel. Shapiro-Wilk test was used to analyze the normality of data. ANOVA and Student’s t-tests were used to compare groups. All tests performed were two-sided with an alpha level of 0.05. A p-value of <0.05 was considered statistically significant. All data are presented from either three or five independent experiments as indicated, with mean ± standard error (SEM).

## Results

### Effect of H_2_O_2_ treatment on viability and intracellular oxidative stress of PG and SMG cells

**FIGURE 3:**
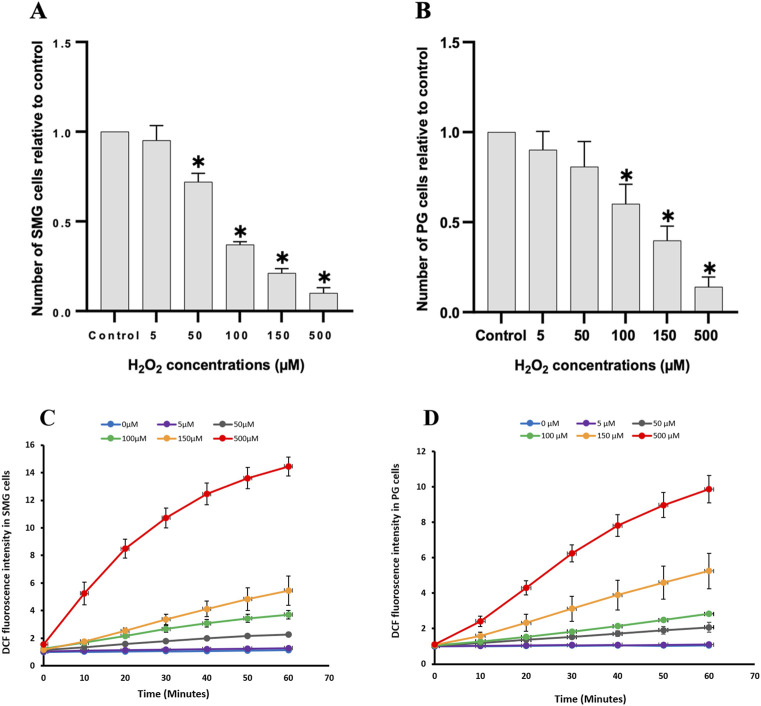
Cell viability and intracellular reactive oxygen species were measured in PG and SMG acinar cells. Cell viability was measured with Trypan blue in **(A)** SMG and **(B)** PG cells treated with different concentrations of H_2_O_2_ (n = 5) and presented relative to the unstimulated control cells. *p-value <0.05 for each treated group compared to a control group. Intracellular reactive oxygen species measured with the CM-H2DCFDA fluorescence assay in **(C)** SMG and **(D)** PG cells treated with different concentrations of H_2_O_2_ (n = 3) for 60 min and presented relative to the unstimulated control cells. Each color represents a different H_2_O_2_ concentration.

To assess the impact of H_2_O_2_ on cell viability, we counted the number of live cells after 24 h treatment and compared them to control (Figure 3). Increased H_2_O_2_ concentration led to a significant reduction in cell viability for both SMG and PG cells (Figures 3A, B, respectively). There was a clear dose-response relationship, with higher H_2_O_2_ concentrations consistently associated with lower cell viability. To evaluate whether this reduction in viability corresponded with a general increase in oxidative stress, we also measured the level of intracellular ROS.

We used the CM-H2DCFDA fluorescence assay to examine the impact of various H_2_O_2_ concentrations on ROS levels in PG and SMG cells. The ROS concentration increased steadily in a dose-dependent manner during the 60 m of the experiment, in both SMG (Figure 3C) and PG cells (Figure 3D).

### Effect of H_2_O_2_ treatment on gene expression of autonomic receptors and SOCE components in PG and SMG cells

**FIGURE 4:**
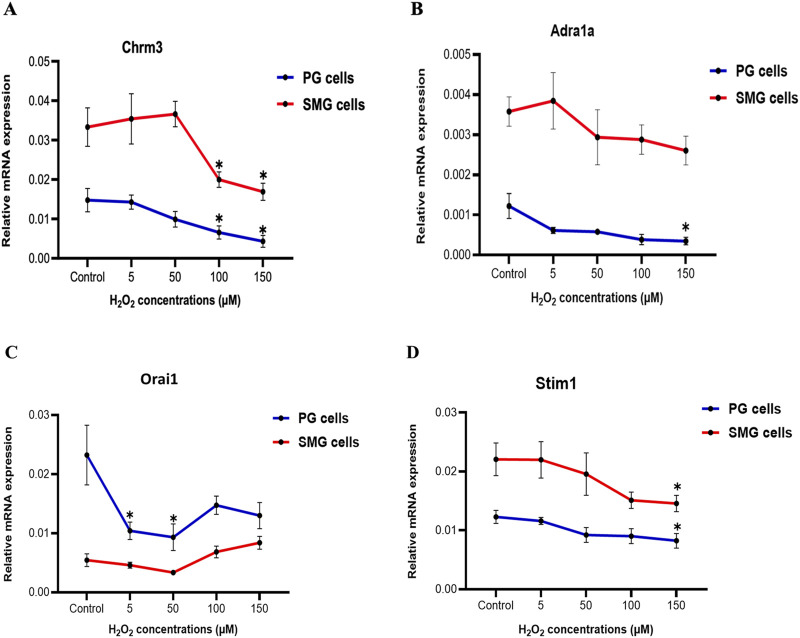
*Chrm3* and *Adra1a, Orai1, and Stim1* gene expression in PG and SMG acinar cells. *Chrm3* **(A)**, *Adra1a* **(B)**, *Orai1* **(C)** and *Stim1* **(D)** mRNA levels were analyzed by RT-qPCR in cells exposed to varying H_2_O_2_ concentrations. Gene expressions were normalized to the housekeeping gene *Rpl27*. Relative expression from five independent experiments. *p-value<0.05 for each treated group compared to control.

We examined how H_2_O_2_ influenced the expression of certain genes in the intracellular Ca^2+^-signaling pathway that regulates primary saliva production in acinar cells. We observed a substantial and significant downregulation of the cholinergic receptor muscarinic 3 subtype (*Chrm3*) mRNA expression in both PG and SMG cells when exposed to elevated H_2_O_2_ concentrations (100, 150 µM) in comparison to the control group (Figure 4A). For the alpha-1A adrenergic receptor (*Adra1a*), there was a slight reduction in both SMG and PG cells (Figure 4B). However, this reduction was only statistically significant in PG cells exposed to 150 µM H_2_O_2_. Interestingly, we found that the expression of both receptors was significantly higher in SMG compared to PG cells.

Next, we examined the mRNA expression of *Stim1* and *Orai1*, which are components of the SOCE mechanism crucial for saliva production. Figure 4C shows a significant downregulation of *Orai1* mRNA expression at 5 and 50 µM H_2_O_2_ in PG cells, followed by a slight, but not significant increase at higher concentrations. In contrast, in SMG cells, mRNA expression of *Orai1* showed no significant changes. Furthermore, there was a gradual decline in *Stim1* mRNA expression in both PG and SMG cells with increasing H_2_O_2_ concentrations, but this reduction was significant only at 150 µM for both cell lines (Figure 4D).

### Effect of high doses of H_2_O_2_ on Orai1 and STIM1 protein expression in PG and SMG cells

We conducted Western blotting to assess the protein expression levels of Orai1, STIM1, Chrm3, and Adra1a in PG and SMG cells exposed to 100 and 150 μM H₂O₂. Additionally, we used immunofluorescence microscopy to evaluate the expression of Orai1 and STIM1 in PG and SMG cells exposed to the same H₂O₂ concentrations.

**FIGURE 5:**
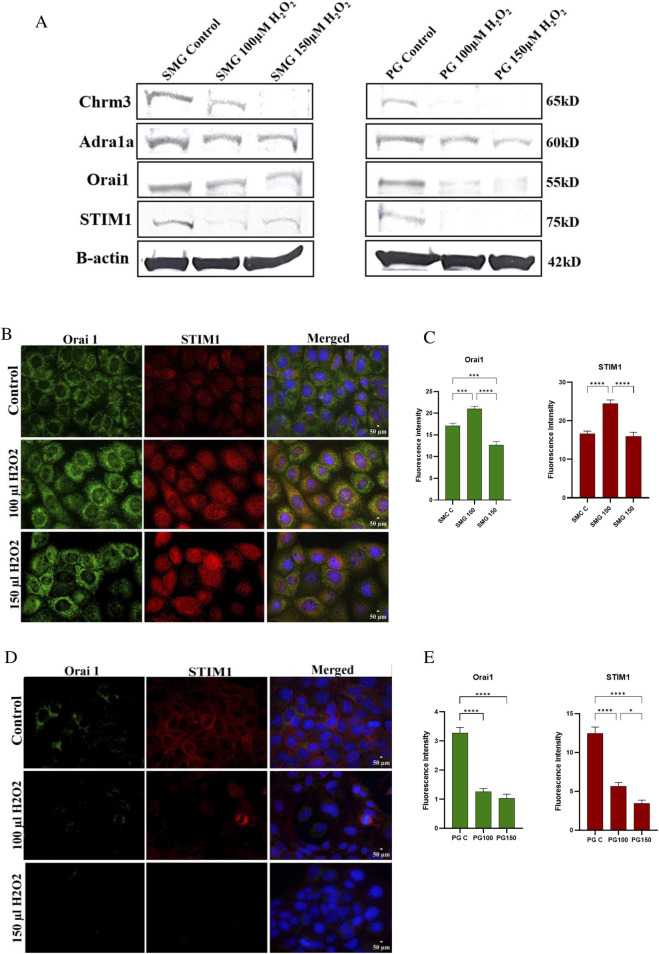
Chrm3, Adra1a, Orai1, STIM1 protein expression in SMG and PG acinar cells. Western blot analysis was conducted to measure protein expression of Chrm3, Adra1a, Orai1 and STIM1 **(A)** in SMG and PG cells exposed to 0 (control), 100 and 150 μM H₂O₂. B-actin used as a reference protein. Orai1 and STIM1 protein expression in SMG and PG acinar cells were assessed by immunofluorescence microscopy, and their fluorescence intensities were measured in SMG **(B, C)** and PG **(D, E)** cells exposed to 0 (control), 100 and 150 μM H₂O₂. DAPI was used for nuclear staining. All photos were taken with the same exposure time (500 ms), analog gain (9.3x), and 40x magnification. Representative pictures from three independent experiments.

Protein expression of Chrm3 was downregulated by H₂O₂ in both SMG and PG cells (Figure 5A). For Adra1a, protein expression decreased in PG cells with increasing H₂O₂ concentrations, whereas in SMG cells, H₂O₂ exposure did not produce notable changes in protein expression compared to the control. Additionally, Orai1 and STIM1 expression were reduced in PG cells following exposure to H₂O₂. In SMG cells, Orai1 expression was comparable to the control when H₂O₂ levels were increased, while STIM1 expression decreased, with a more pronounced reduction at 100 μM H₂O₂ than at 150 μM H₂O₂.

The immunofluorescence microscopy showed somewhat dissimilar effects of H_2_O_2_ on protein staining in the SMG cells compared to western blot. Both Orai1 and STIM1 staining intensity was increased at 100 µM. At 150 µM H_2_O_2,_ Orai1 was reduced compared to control cells while STIM1 was similar to control levels (Figures 5B, C). For the PG cells, immunofluorescence and western blot results were consistent, the protein staining of both Orai1 and STIM1 showed a reduction in intensity in PG cells when exposed to both H_2_O_2_ doses (Figures 5D, E).

## Discussion

Hyposalivation, a condition of reduced saliva production that significantly affects oral health, may be caused by ROS-induced oxidative stress in several different situations (Villa et al., 2015; Checa and Aran, 2020). For instance, it has previously been demonstrated that the accumulation of endogenous ROS in irradiated human salivary gland cell lines leads to increased oxidative stress and impaired Ca^2+^ signaling, which are associated with reduced salivary gland function (Liu et al., 2017). Increased oxidative stress has also been implicated in Sjögren’s syndrome (Ryo et al., 2007), where the Ca^2+^ signaling is similarly impaired (Enger et al., 2014). Therefore, our study aimed to investigate specific components of the Ca^2+^ signaling system and their responses to increased oxidative stress in salivary gland acinar cells.

We used H_2_O_2_ as a model of ROS induction, and our data confirmed an increase in ROS, and thus oxidative stress, in both PG and SMG cells with increasing H_2_O_2_ concentrations, which is reasonable given the ability of H_2_O_2_ to diffuse across lipid bilayer as well as through aquaporins (Bienert et al., 2007). Thus, this treatment induced an imbalance between ROS production and the antioxidant defense mechanisms, resulting in increased oxidative stress. This imbalance may in turn lead to cellular dysfunction, cell damage, and even cell death (Murphy et al., 2011; Sies and Jones, 2020), which *in vivo* is likely to present clinically as hyposalivation (Niki, 2016; Sies, 2017). As expected, our results showed a reduced viability of both PG and SMG cells when the H_2_O_2_ concentration increased. It is important to highlight that the parotid gland contains serous acini, whereas the submandibular gland contains both mucous and serous acini (Proctor, 2016). Thus, the two cell lines may well display differences in sensitivity to oxidative stress when it comes to viability and cell survival. This may be interesting to pursue in later studies.

Further, we investigated the effect of H_2_O_2_ on central components of the fluid secretion signaling system, first by measuring mRNA expression of the acetylcholine receptor (*Chrm3*) and alpha-1A adrenergic receptor (*Adra1a*), linked to the parasympathetic and sympathetic systems, respectively. The decreased expression of these receptors with increasing H_2_O_2_ levels suggests that H_2_O_2_-induced oxidative stress can have an inhibitory effect on the acinar cell response to the autonomic nervous system and may thus affect the regulation of saliva production. Furthermore, western blot analysis confirmed a reduction in Chrm3 and Adra1a protein expression in both SMG and PG acinar cells following H₂O₂ exposure. However, Adra1a protein levels appear to be less affected by H₂O₂ in SMG cells compared to PG cells, and less affected than Chrm3 in both cell lines. Reduced expression of *Chrm3* has also been observed in both diabetic and irradiated rat salivary cells compared to control (Chen et al., 2020; Wu et al., 2021), and we may speculate that this reduction is due to increased oxidative stress. To the best of our knowledge, this is the first time a reduced expression of any autonomic receptors during H_2_O_2_-induced oxidative stress is demonstrated. Our results reveal that the effects of oxidative stress on PG and SMG cells are not limited to parasympathetic and sympathetic receptors, emphasizing the necessity of considering also downstream components of the Ca^2+^ signaling pathway. We found a reduction in both mRNA and protein levels of STIM1 and Orai1 in the PG cells when exposed to elevated concentrations of H_2_O_2_. In contrast, in SMG cells, high H₂O₂ concentrations induced a significant reduction in *Stim1* mRNA expression and an increase in *Orai1* expression, although this increase was not significant. Protein expression of STIM1 and Orai1 appeared to increase at 100 μM H₂O₂. However, at 150 μM H₂O₂, Orai1 expression decreased compared to control levels, while STIM1 expression remained comparable, as indicated by immunofluorescence staining. In Western blot analysis, Orai1 expression remained similar to control levels with increased H₂O₂, whereas STIM1 expression showed a decrease, more pronounced at 150 μM H₂O₂ than at 100 μM H₂O₂. We speculate that difference between immunofluorescence staining, and Western blotting may arise from reduced cell counts at 150 μM H₂O₂, potentially leading to an underestimation of fluorescence intensity due to fewer cells available for staining. Additionally, nonspecific antibody binding may vary across conditions, potentially affecting fluorescence intensity and resulting in apparent changes that may not accurately reflect actual protein expression levels.

Moreover, Liu X et al. have shown that radiation induces a decrease in STIM1 protein expression in SMG acinar cells in mice (Liu et al., 2017; Liu et al., 2021). The different effects of H_2_O_2_ on mRNA and protein expression, respectively, on STIM1 in SMG cells may indicate that the effect is due to post-transcriptional or translational processes. It has been demonstrated that both Orai1 and STIM1 undergo several types of post-translational modifications, including redox modifications induced by ROS, which may influence the stability of the proteins and thus SOCE, as reviewed by Johnson et al. (2022). It can be speculated that the increasing effect on protein levels may be attributed to redox modifications counteracting the reducing effect on transcription. In addition, it may be speculated that the increased protein expression of STIM1 in SMG cells may reflect a cellular response aimed at protecting or enhancing SOCE activity in response to oxidative stress.

There are several studies demonstrating that ROS affects SOCE in other cell types, similar to our findings in salivary acinar cells. For instance, H_2_O_2_ reduces SOCE current in prostate cancer cell lines (Holzmann et al., 2015) and human platelets (Redondo et al., 2004), and increases mRNA expression of *Orai1* and *Stim1* in bovine brain capillary endothelial cells (Yamamura et al., 2020). Interestingly, the SOCE current as well as Orai1 and STIM1 protein expression decreases when intracellular pH decreases in HEK293T human embryonic kidney cells (Rychkov et al., 2022). Since H_2_O_2_ induces an intracellular pH reduction (Kaufman et al., 1993), changes in intracellular pH may be another pathway for the observed reduction in STIM1 and Orai1 expression under high H_2_O_2_ exposure in the PG cells. These findings underscore the varied impacts of H_2_O_2_-induced oxidative stress on Ca^2+^ signaling components across different cell types.

Our study demonstrated different responses to oxidative stress between the PG and SMG rat cell lines under the same conditions. To the best of our knowledge, there are no other studies showing such a differential response to oxidative stress. On the other hand, several clinical investigations have demonstrated that the parotid glands are more radiosensitive than the submandibular glands (Liem et al., 1996; White, 2009), at least regarding the acute effect of irradiation. This fits with the hypothesis presented by Liu et al. (2017) and others (Ambudkar, 2018) that the acute hyposalivation observed after radiation treatment may be due to increased ROS in the acinar cells, leading to reduced SOCE activity. It is well known that irradiation elevates ROS levels and triggers oxidative stress (Checa and Aran, 2020), thus our observations of more substantially reduced Orai1 and STIM1 levels in PG cells relative to SMG cells when exposed to H_2_O_2_ are consistent with the documented higher radiosensitivity of the parotid gland compared to the submandibular gland.

## Conclusion

In conclusion, our study showed that H_2_O_2_ exposure induces changes in PG and SMG rat acinar cell lines, including decreased expression of key autonomic receptors. Different impacts on STIM1 and Orai1 expression between these cell lines highlight the complex response of the Ca^2+^ signaling system to oxidative stress. More research is needed to explore the impact of oxidative stress on SOCE and other Ca^2+^ signaling components. A deeper understanding of ROS-induced damage to the salivary glands may provide future strategies to protect and preserve salivary gland cells against oxidative stress.

## Data Availability

The raw data supporting the conclusions of this article will be made available by the authors, without undue reservation.
